# A synthesis of convergent reflections, tensions and silences in linking gender and global environmental change research

**DOI:** 10.1007/s13280-016-0843-0

**Published:** 2016-11-22

**Authors:** Irene Iniesta-Arandia, Federica Ravera, Stephanie Buechler, Isabel Díaz-Reviriego, María E. Fernández-Giménez, Maureen G. Reed, Mary Thompson-Hall, Hailey Wilmer, Lemlem Aregu, Philippa Cohen, Houria Djoudi, Sarah Lawless, Berta Martín-López, Thomas Smucker, Grace B. Villamor, Elizabeth Edna Wangui

**Affiliations:** 1 Social-Ecological Systems Laboratory, Department of Ecology, Edificio de Biología, Calle Darwin nº 2, Universidad Autónoma de Madrid (UAM), Campus de Cantoblanco, C.P. 28049 Madrid, Spain; 2 ICAAM - Instituto de Ciências Agrárias e Ambientais Mediterrânicas, LDSP - Landscape Dynamics and Social Process Research Group, Universidade de Évora, Pólo da Mitra, Ap. 94, 7002-554 Évora, Portugal; 3 CREAF, Cerdanyola del Vallès, 08193 Catalonia, Spain; 4 School of Geography and Development and Udall Center for Studies in Public Policy, University of Arizona, 803 East 1st Street, Tucson, Arizona 85719 USA; 5 Internet Interdisciplinary Institute, Av. Carl Friedrich Gauss 5 Open University of Catalonia, 080193 Castelldefels, Barcelona Spain; 6 Institut de Ciència i Tecnologia Ambientals, Edifici Z ICTA-ICP Carrer de les columnes, Universitat Autònoma de Barcelona, Bellaterra, 08193 Cerdanyola del Vallès, Barcelona Spain; 7 Department of Forest & Rangeland Stewardship, Colorado State University, 1472 Campus Delivery, Fort Collins, CO 80523-1472 USA; 8 University of Saskatchewan, 117 Science Place, Saskatoon, SK S7N 5C8 Canada; 9 International START Secretariat, 2000 Florida Avenue N.W., Suite 200, Washington, DC 20009 USA; 10 Worldfish, West Gyogone, Bayint Naung Road, Insein Township, Yangon, 11181 Myanmar; 11 WorldFish, Jalan Batu Maung, Batu Maung, 11960 Bayan Lepas, Penang Malaysia; 12 ARC Centre of Excellence for Coral Reef Studies, James Cook University, Townsville, Queensland 4811 Australia; 13 CIFOR, Centre for International Forestry Research, Situ Gede, Bogor Barat, Jawa Barat 16115 Indonesia; 14 Faculty of Sustainability, Institute of Ethics and Transdisciplinary Sustainability Research, Leuphana University, Scharnhorststr. 1, 21335 Lüneburg, Germany; 15 Department of Geography, Ohio University, Clippinger Labs 122, Athens, OH 45701 USA; 16 Center for Development Research (ZEF), Walter-Flex 3, 53113 Bonn, Germany

**Keywords:** Feminist political ecology, Global environmental change, Intersectionality, Reciprocity, Reflexivity

## Abstract

This synthesis article joins the authors of the special issue “Gender perspectives in resilience, vulnerability and adaptation to global environmental change” in a common reflective dialogue about the main contributions of their papers. In sum, here we reflect on links between gender and feminist approaches to research in adaptation and resilience in global environmental change (GEC). The main theoretical contributions of this special issue are threefold: emphasizing the relevance of power relations in feminist political ecology, bringing the livelihood and intersectionality approaches into GEC, and linking resilience theories and critical feminist research. Empirical insights on key debates in GEC studies are also highlighted from the nine cases analysed, from Europe, the Americas, Asia, Africa and the Pacific. Further, the special issue also contributes to broaden the gender approach in adaptation to GEC by incorporating research sites in the Global North alongside sites from the Global South. This paper examines and compares the main approaches adopted (e.g. qualitative or mixed methods) and the methodological challenges that derive from intersectional perspectives. Finally, key messages for policy agendas and further research are drawn from the common reflection.

**Unsolicited Advice**

*Embrace complexity.*

*Tolerate ambiguity.*

*Take risks.*

*Work hard.*

*Give back.*

*Help each other out.*

*Follow through.*

*Practice reflexivity*

*Reflect on the process as well as the outcome,*

*The relationships as well as the products*

*Do it as a team.*

*Seek out your toughest critics.*

*Take a hard look at yourself.*

*Own your privileges and know your biases.*

*Don’t take yourself so seriously.*

*Be vulnerable.*

*Be strong.*

*Be generous.*

*Be a transdisciplinary woman warrior.*

*Take care of your body, heart and spirit,*

*While you cultivate your mind.*

*Speak truth to power*

*(But only after you have tenure).*

*Know that your truth is your power.*

*Love what you do.*

*Do what you love.*

*Do more.*

*Do less.*

*Pay it forward.*

*Don’t drop the ball.*

*Learn to drop the ball.*

*Go for a walk every day.*

*Write poetry.*

*Laugh.*

*Laugh more.*

*Now laugh like there is joy in you.*

*Help each other out.*

*Help each other out.*

*Help each other out.*

*Give back.*

*Pay it forward.*

An earlier version of this poem was written by María E. Fernández-Giménez and it was included in a keynote talk she gave at the Symposium on Interdisciplinary Perspectives on Environmental Change organized by the Graduate Women in Geography at San Diego State University, 26 February 2016. The poem was then enriched by the collaboration with Maureen Reed. The poem reflects on the gender dimensions of interdisciplinary science within academia, and specifically the ethic of reciprocity and practice of reflexivity that women have brought to research teams they have been part of, and which contribute to our collective learning, individual self-actualization and scientific success, as well as ensuring that our research directly benefits the communities where we work.

## Reflexivity and reciprocity in research processes

**T**his synthesis article aims to join all the authors in a common reflective dialogue on the main contributions and research process of their papers, linking gender and feminist approaches to research in adaptation and resilience in global environmental change (GEC). Such a process derives from the practice of reflexivity, common in feminist studies. Feminist and critical scholars from many disciplines have long been aware of how the politics of their multiple positions affect field work and research processes more broadly (McDowell 1992; England 1994; Kobayashi 1994; Rose 1997; Rocheleau 2015; Faria and Mollett 2016). Reflexivity has been described as a key feminist research practice used to locate oneself in the research process and to foster scholars’ reflection regarding how knowledge is situated and shared, how power relations have an impact in the research and researchers, as well as in generating and circulating knowledge (Rose 1997; Reed and Peters 2004). In terms of GEC, when reflexivity is practiced by academic and community researchers, it can help them jointly build an agenda for action research that helps communities identify vulnerabilities, consider adaptive strategies and build resilience in the face of immediate threats and long-term challenges.

If reflexivity is the major theme of the poem, a second important theme is reciprocity. The authors of the poem (and many in this special issue) adhere to an ethic of reciprocity in our relationships with our “subject” communities. This commitment to reciprocity flows from the practice of reflexivity, which cultivates awareness of the power dynamics embedded in the research process and motivates us as researchers to counteract asymmetrical or extractive relationships when we identify them (Smith 1999). As “outsiders”, we can do this by demonstrating long-term commitment to local research partners, pursuing questions they identify, engaging with them as co-researchers, ensuring that results are returned to their community in a form they can understand and use, co-authoring with community partners, and offering training and capacity building. We also seek to instil an ethic of reciprocity within our research teams, nurturing a culture of collaboration, mutual support, shared learning, and accountability to our team members and community partners.

Thinking in a collaborative reflexive process among the researchers who take part of this special issue, this synthesis is structured around key questions the guest editors posed to the authors (Table 1). The result is a text where the voices of authors are highlighted in italics in an attempt to record their own experiences and reflections. The paper is thus organized around these five broad themes: (1) theoretical reflections on tensions and convergences linking GEC literature and resilience theories with gender and feminist studies; (2) empirical insights that further develop the analysis of marginalization and vulnerability to include agency and valorize the importance of invisible spaces; (3) methodological advances; (4) multi-scalar policy and programmatic actions and (5) suggested ways ahead for the research agenda.

**Table 1:** Questions posed by the guest editors to the authors in a reflexive dialogue

Theme	Questions
Contributions from feminisms to research in adaptation and resilience in GEC	
Theoretical contributions	Why have you used a specific theoretical approach and why now? How do you assess the combination of different theories (e.g. gender/feminist theories/adaptation, resilience theories) from the theoretical standpoint taken in your paper? Have they enriched each other? Do you think one contributed more to the other? Are there ways in which they do not fit?
Advances in methodologies	Which are specific methodological challenges (frameworks, methods, tools) in combining gender/feminist theories and adaptation, resilience and vulnerability research that you’ve found? Why have the methodologies applied in your paper been more useful than other methodologies to tackle gender in adaptation and resilience to GEC? How was your paper able to ‘translate’ the gender questions to local communities? What were the risk involved and what were the failures? (e.g.. Failure because often researchers shy away or hide the difficulties in conduction ‘new’ advanced methodologies)
Other reflections	
	Have you come up with other reflections in working at the intersection of gender and adaptation, resilience to GEC? Which are the obstacles you have found so far in the application of feminists theories and movements on adaptation and resilience to GEC?
Key messages for policy and programming in respect to GEC	
	What are your key messages and key targets for programmes and interventions in research and policy agendas on GEC? Which are the enabling factors that can support the gender agenda in GEC policy?
Ways ahead/further research	
	Which and how diverse feminist theories and movements may dialogue and contribute further to resilience and adaptation research in GEC? Which avenues should be taken into account and further explored in combining gender/feminist research and adaptation and resilience to GEC?

## Theoretical reflections

The authors who participated in this special issue engage the diverse disciplines of Geography, Anthropology, Rural Sociology, Development studies, Environmental, Resilience, Gender and Feminist studies. They contribute from different understandings to the feminist political ecology literature and gender studies in environmental management and conservation, by finding a space for dialogue that connects social and political dynamics with the ecological system. To respond to the demand for interdisciplinary and comprehensive work that links gender and GEC, the authors push forward the boundaries of theories and empirical research to reflect on issues of power in gendered environmental analysis and to combine ecological theories of resilience in social analysis. The main theoretical contributions of this special issue rely on emphasizing the relevance of power relations in feminist political ecology, bringing the livelihood and intersectionality approaches into GEC, and linking resilience theories and critical feminist research.

Some of the papers included in the special issue focus on responding questions of classical feminist political ecology by understanding the underlying resilience and adaptation processes for facing GEC and the vulnerability of particular social agents. On the one hand, women’s and men’s everyday practices shape their knowledge and understanding related to environmental change. On the other hand, this knowledge interacts with complex power structures (including institutional contexts and processes within which they are embedded) to shape how women and men perceive their vulnerability and their capacity to respond (Bee 2016). Therefore, feminist political ecology understands issues of power as the result of these everyday practices that influence unequal social relations and the response to GEC.

As Buechler (2016a) suggests: *feminist political ecology allowed for an analysis of how gender and social class influenced the locus of agricultural production, which in turn shaped the nature of climate and water*-*related vulnerabilities experienced and grassroots responses to these. Feminist political ecology and the concept of adverse inclusion helped to shed light on subjectivities linked to policies and programs*. The concept of ‘adverse inclusion’ is applied in Northwest Mexico by Buechler (2016a) to examine local vulnerabilities and community-level adaptation and to reflect on the issue of scale. According to Buechler: *scalar issues emerged from an analysis that started in and around the home, which continues to be an important locus for women in rural Mexico, and indeed, for women globally and extended to community and regional levels*. In fact, feminist political ecology allows to understand the different roles, responsibilities and power relations across scales: from global to sub-community level (Rocheleau et al. 1996; Elmhirst 2011).

To tackle social differentiation at “sub-community” levels, other theoretical frameworks and approaches, such as the livelihood approach from development studies, are also proposed to analyse individual and collective adaptive capacity. According to Philippa Cohen and colleagues in the analysis of agriculture and fisheries dependent communities in Solomon Islands (Cohen et al. 2016): *we apply aspects of gender theory to assess individual capacities to innovate and adapt in social*-*ecological systems through a framework that defines five dimensions of adaptive capacity: assets, flexibility, learning, social organisation and agency*. Such a framework examines the opportunities and constraints for women and men take on to construct and modify their livelihood in response to GEC (e.g. Scoones 1998; Bebbington 1999). The same framework also allows for the expansion of the adaptive capacity literature at the household and community level to consider the intersections across units of analysis. In the implementation of the livelihood framework to the case study of Sami reindeer husbandry in northern Sweden (Buchanan et al. 2016), Maureen Reed, co-author of the paper, highlights: *the analyses must examine multiple scales simultaneously*—*so not just intersections across the same scale (e.g., individual), but across scales (individual, household, community, and region) as there are interplays among scales.*

Several papers in this special issue also explore the power issues from an intersectionality perspective. According to such a perspective, multiple social identities and forms of oppression, such as class, race, ethnicity, caste, sexuality and age intersect and influence environmental management, livelihood vulnerability and adaptation responses to GEC (Nightingale 2011; Kaijser and Kronsell 2014). From the experiences documented in this special issue (e.g. Djoudi et al. 2016; Ravera et al. 2016), it is highlighted that, both in praxis and in theory, the mainstreaming gender framework and analysis as it is applied in science, specifically in climate change adaptation, is misleading, meaning there is a risk that existing inequalities will be ignored and/or exuberated. In fact, there is a risk of not considering differences within gender groups due to multiple and hidden underlying factors of inequity in access, use and management of resources in the context of GEC. Mary Thompson-Hall concludes: *more and more practitioners, decision makers and researchers are acknowledging that the explanatory power of pre*-*conceived assumptions around gender and vulnerability are limiting effective adaptation strategizing.* Intersectionality, thus, as resumed by Houria Djoudi: *helps to overcome the simplified dichotomy of “men” versus “women” in the gender analysis of climate change and the view of women as unitary subjects*. *It also helps to draw attention that vulnerability is not something we are born with because we are women. Vulnerability is related to the participation in decision making, access to resources, voice, which are all related to the positioning of individuals in their society or community. (…) Rather than creating a “vulnerability Olympics”: looking for who is the most vulnerable, it helps to understand the root causes of inequities and vulnerability*.

In summary, by disputing pre-defined categories and positioning individuals in the context of power relations, the intersectionality approach may refine, unpack and enrich the understanding of vulnerability, resilience and adaptation to GEC.

Three other empirical papers apply the gendered lens to resilience theory and empirical research (Aregu et al. 2016; Díaz-Reviriego et al. 2016; Wilmer and Fernández-Giménez 2016). As suggested by Thomas Smucker: *by bringing a gendered lens to established concepts in the climate change lexicon (local knowledge, resilience, adaptation), we can contribute to refining our understanding of those terms and avoiding the use of simplistic metaphors derived from the natural sciences to describe processes that are complex, heterogeneous, and reflect dynamics of gender inequality.* Developing further the idea of gender inequality, Hailey Wilmer recognizes a tension between resilience theories and critical feminist research highlighting that: *the state of things for women, however, is not a place we want to stay. Resilience, then, is too often framed as a positive attribute of a system that implies social equality, but conceptually blinds unequal relations in everyday practices within the house and the community*. In this sense, Wilmer and Fernández-Giménez (2016) conceptually define resilience as an embodied practice by adopting a cultural resilience approach (Crane 2010) in their research on rangeland systems in Southwestern United States. As explained by Hailey Wilmer: *we wanted to highlight the resistance of women to interconnected biophysical, economic and cultural sources of oppression, and push*-*back against static or romantic representations of ranching women. (The) biophysical scientists are more interested in learning about rancher decision*-*making in these extensive beef production systems, (but they) often overlook power dynamics that drive decisions on a family farm*.

Díaz-Reviriego et al. (2016) and Aregu et al. (2016) combine resilience theories with gender analysis to understand the different sources of environmental change that the system needs to adapt and cope with. Isabel Díaz-Reviriego and co-authors explicitly argue that the integration of the gender analysis with different ecological theories might make: *visible uneven knowledge distribution, and specifically gendered knowledge, within communities in analysing the resilience of local medical systems*. Similarly, Lemlem Aregu highlights that: *gender analysis enriches the resilience analysis by informing how gender inequality contributes to change the existing social*-*ecological system due to gendered roles in access to and control over natural resources and its management rules. (…) Moreover, resilience analysis will enrich gender by asking the question of how priorities, experiences and adaptation capacity in the face of shocks are shaped by gender inequalities and vice versa*.

## Empirical insights

The papers of the special issue also provide empirical evidence on particular debates in GEC research, namely vulnerability vs. resilience, and women vs men dichotomies. Further, the special issue also contributes to broaden the gender approach in adaptation to GEC by incorporating new research sites (i.e. Global North) and spaces (i.e. orchards) and by drawing socio-ecological consequences of gender inequality.

First, some papers contest the “impacts narrative” as the central argument in GEC debates in favour of overcoming dichotomies that oppose vulnerability, adaptation and resilience concepts (Tschakert and Tuana 2013). Buechler (2016a, b) provides an example of such insight in her case study in Northwest Mexico. As she observes: *the spaces that women and men farm are different due to gendered land ownership and the type of water available (…). In San Ignacio (Sonora, Mexico), women farmers innovate with respect to irrigation water sources, by reusing water and by combining more than one source, and engage in crop diversification within home garden production. Men with small land parcels have adapted through crop diversification and sharing crops with wildlife pushed out of their habitats. Men are also engaging in adaptation strategies like adding on*-*farm and non*-*farm income sources and renting orchards to urban residents.*

Second, particular empirical papers contest the narrative of women vs. men (e.g. Ravera et al. 2016) and contribute with new insights to the exploration of underlying contextualized factors that define inequity power relations, by interrogating the intersections of different social categories (for similar studies, see Carr 2008; Onta and Resurreccion 2011; Van Aelst and Holvoet 2016). In the words of Federica Ravera: *the time is ripe for intersectionality and feminist studies to serve as strong connecting pieces between the different bodies of knowledge related to GEC*. In fact, by observing intra-community gendered responses in different social-ecological contexts in India, Ravera et al. (2016) empirically demonstrate how, even with similar GEC and other multiple agrarian stressors, decisions and new roles and relations of women and men within the household and the community are differently renegotiated, depending on the context, the livelihoods, the class and the caste, catalysing or constraining adaptation among groups.

Finally, regarding gendered subjects and gender, the focus of some of the papers in the special issue (Buchanan et al. 2016; Wilmer and Fernández-Giménez 2016) opens the question of gender in adaptation, vulnerability and resilience in GEC to subjects located in the Global North. While maintaining its rural background, these papers broaden the (previously) almost exclusive focus on women in developing countries (MacGregor 2010). Further, some papers in the special issue do not only look for material aspects of gender but also extend it to its discursive dimensions (Wilmer and Fernández-Giménez 2016), taking into account that gender is not just an empirical category or identity but it also is a discursive, social construction that organizes the world (MacGregor 2010). By using the framework suggested by O’Shaughnessy and Krogman’s (2011), Wilmer and Fernández-Giménez (2016) open room for material and discursive analysis of gendered practices by exposing the contradictions between discourse in ranching culture and women’s material practices.

Additionally, Aregu et al. (2016) show evidence of how gender relations extend and configure the ecological system. As Lemlem Aregu says: *this focus helps us to understand how not including the different interest and preferences of women can shape the natural resources dynamics which is the domination of unpalatable species in the pasture. Exclusion of women from the informal institution also inhibits adaptive capacity of the system to respond to the spread of the unpalatable species. If women participated in the decision*-*making process of rule crafting, provision of women access to harvest that specific grass species, could be the possible adaptation measures to maintain the quality of the pasture at the same time fulfil women’s interest to craft the basket using the grass.*

## Methodological advances

Special issue authors reflect on methodological challenges related to (1) qualitative, (2) applying mixed-methods approaches and (3) methodological challenges that derive from intersectional perspectives.

### Challenges of qualitative approaches

Qualitative methods have had a stronger presence in feminist research because they explicitly pose epistemological questions regarding how knowledge is generated, by and for whom. Further, they challenge the claims of objectivity and neutrality made by the vast majority of researchers working with quantitative methods (Smith 1999; Nightingale 2003; O’Shaughnessy and Krogman 2012; Adams 2014). In fact, qualitative methods can address power and representation based on the feminist principles of respecting women’s and other oppressed groups’ unique ways of knowing, seek to address the colonial and heteropatriarchal nature of knowledge by giving voice to the everyday experiences of research participants, and confront socially constructed gendered inequalities (Haraway 1988; Alcoff 1991; Islam 2001; Hesse-Biber 2007; Adams 2014). Feminist research methodologies have sought to re-imagine researcher–participant relationships so as to build bridges across disciplines, and across serial categories of class, race, ethnicity, gender and ability.

Regarding qualitative methodologies used in the collection of articles of this special issue, interviews and focus groups were among the most common qualitative methods used (Aregu et al. 2016; Buechler 2016a; Cohen et al. 2016). This is consistent with the results found by O’Shaughnessy and Krogman (2012) regarding qualitative methods used in feminist research. In addition, this special issue also contributes by applying innovative methodological tools in GEC, such as narrative and ethnographic approaches.

Narrative approaches like oral histories (used by Wilmer and Fernández-Giménez 2016) are methods extensively used in feminist research but infrequent in adaptation and resilience to GEC (e.g. Hanson 2015, 2016). Oral histories have been used by feminists to tell alternative histories and to present multiple perspectives by interpreting the values, symbols and contradictions contained in individual accounts. These narratives link the past with the present through the words and experiences of the individuals telling them (Nightingale 2003). As explained by Hailey Wilmer, narrative methodologies aim: *to create the research through an iterative, collaborative process that can enrich the findings and the value of the product to the various parties. In this kind of work, the subjectivity of the researcher is also at play in the research process. The challenge is to do good, objective work that honours the research participants and seeks to present their voices accurately, while recognizing the limitations of the knowledge and the role of one’s own experience in shaping the work*.

Buechler (2016a) uses a long-term, ethnographic approach, which allows for an analysis of coping strategies versus more sustainable strategies that women and men engage into address environmental change. A shorter-term study would not have revealed the dynamism in the interactions between the producers and their environment, especially in terms of innovation and experimentation in cropping patterns and water management. The importance of long-term research is also emphasized by Philippa Cohen and colleagues: *in a temporal study a researcher can observe how latent capacities [to adapt] play out [in response to change], are facilitated or hindered, and how gender norms and relations are influential and influenced*. Indeed, field visits over time become akin to visits with old friends in a process that entails discovering what has happened in the producers’ lives especially in terms of environmental change and their strategies to deal with this change (Buechler 2016b). However, the long-term ethnographic approach faces some challenges, particularly those related with the time and effort needed to maintain ties with key interviewees. This is even more challenging when the gender perspective is considered as each field visit should have some time to talk to women separately from male family members in order to be able to ensure that women’s experiences are shared.

### Challenges of the mixed-methods approach

The mixed-methods approach, which aims at combining techniques for collecting qualitative and quantitative data, has gained considerable traction in feminist research as a means of balancing the drawbacks of each technique (Nightingale 2003; Leckenby and Hesse-Biber 2007; Buechler and Hanson 2015). In this special issue, the mixed-methods approach are also used in half of the case study papers (Buchanan et al. 2016; Dah-gbeto and Villamor 2016; Díaz-Reviriego et al. 2016; Ravera et al. 2016; Smucker and Wangui 2016). The value of triangulating of the mixed-methods approach has been emphasized by feminist political ecologists because such approach is able to capture the gendered differences in access to, control over and knowledge of resources (Rocheleau 1995; Nightingale 2003; Buechler and Hanson 2015). As explained by Isabel Díaz-Reviriego: *to combine gender theories with resilience research, we employed mixed methods in trying to achieve a more nuanced understanding of the context of the production of this same knowledge, as well as adaptive capacity and resilience of the local medicinal knowledge system.*

Challenges sometimes arise when employing mixed methods because of epistemological foundations of the different fields (Miller et al. 2010). As Mary Thompson-Hall describes: *at times it has seemed difficult to bring together communities of researchers that come from highly quantitative, physical and biological science backgrounds on one side, with those coming from highly qualitative social science backgrounds on the other. Trends toward interdisciplinarity and transdisciplinarity in studying complex social ecological systems are bridging this divide. However, in many instances these groups, including feminist scholars and those working in GEC, still tend to primarily talk and exchange ideas among themselves*.

Additionally, and as suggested by Nightingale (2003, 2016), future studies can explore the value of mixing methods for highlighting that knowledge is partial and that vantage points from different methodologies produce different views of particular processes and events. Thus, mixed methods also help here to challenge ‘dominant’ representations of social-ecological dynamics demonstrating explicitly how they provide only one part of the story.

Finally, Dah-gbeto and Villamor (2016) challenge the representation of nature of GEC, by adopting experimental games in their case study in Northern Benin by introducing *non*-*linear behaviour* (e.g. the weather pattern and drought through throwing of die) and *dynamic* conditions in the system (as the result of game rules and choices of the players). Whatever the consequence of the game produces, the players have to react to the changed conditions and because of the time element, anticipatory adaptation may have developed by the players. Unlike the household survey and focus group discussion, their perception of the GEC remains static, nevertheless, useful to compare with or support for the gaming results.

### Challenges deriving from intersectional perspectives

Methodological issues in implementing intersectionality frameworks have been described as remaining one of the greatest challenges (Valentine 2007; O’Shaughnessy and Krogman 2012; Kaijser and Kronsell 2014). In this special issue, although few studies implement this approach at the local level, Thompson-Hall et al. (2016) illustrate how intersectional approaches do not have to be only used for gathering massive descriptive datasets about local-level populations but instead can be applied to very specific questions around GEC. Continued links between resilience, adaptation and vulnerability theory and intersectional feminist perspectives might provide the opportunity to engage with the transformative and critical methodologies developed in feminist and decolonial traditions. Such bridge-building may be of critical importance now for resilience scholars seeking opportunities to engage with diverse stakeholders and address issues of power long neglected by conventional representations of social-ecological systems (Cote and Nightingale 2012; Olsson et al. 2015).

## Multi-scalar policies and programmatic actions: Key messages

Contributors to this special issue point out a number of key messages and targets for shaping policy agendas on GEC. Among these, two core themes emerge: (1) disconnects in understandings of problems and potential solutions across scales and stakeholder groups; (2) inclusiveness of marginalized voices, knowledge and expertise in GEC policy agenda.

### Different understandings across scales and stakeholder groups

The question of uptake in policy and practice remains a fundamental challenge for research on gender and resilience, adaptation and vulnerability approaches in GEC. As Edna Wangui highlights: *one persistent challenge is that nationally designed adaptation policies and practices receive political and financial support sometimes at the expense of community*-*based adaptation practices. This can be particularly problematic when such national policies and practices conflict with what is already going on at the community. Sometimes this occurs because national actors do not know what is going on in communities, a knowledge gap that researchers can fill.* Further, the very definition of adaptation and resilience adopted in these policies can present challenges for bridging this gap. For example, Nightingale (2015) shows in two very different parts of the world, Scotland and Nepal, a “scale mismatch” between the way resilience is defined in policies, where resilience is framed in terms of sudden shocks and biophysical change with a number of ethnocentric assumptions on local people’s capacities and abilities to access resources and respond to those changes. In contrast, local people define community resilience and livelihood security differently, as rooted in a sense of place, community and family ties and networks. Nightingale (2015) argues that feminist political ecologists can make significant contributions towards exploring and recognizing scale mismatches and how scale is co-opted by local people as a way to both, distinguish themselves from others to the outside world and to engage across scales.

### Towards inclusiveness in GEC policy agenda

The importance of the inclusion of women’s perspectives and voices and also (importantly) those of other marginalized groups in GEC policy agendas came through from many authors. As argued by Edna Wangui: *even at local level, there is potential that gender blind forms of development will be justified in the name of climate change adaptation. As such, there is a need for local voices to be persistent in speaking to the specific risks and livelihood challenges faced by women as a result of climate change… this means that bringing women directly into planning processes will enhance adaptive capacity at the community level.* Likewise, Houria Djoudi emphasizes the relevance of inclusion for developing legitimate and sustainable adaptation strategies: *the effective participation of women and other marginalised groups from the beginning must be prioritised. Policy makers will end up with a better and more endurable product if they actively seek men and women as well as youth and older residents*.

Both of these themes do, however, illuminate some of the complex challenges involved with bringing feminist perspectives to GEC policies and practice. For example, Arora-Jonsson (2014) pointed out that there are certain tensions when gender is introduced in environmental policies because of expectations that gender research needs to present stable categories that policy and practitioners can work with. Therefore, there is a need for collaborative dialogue and, at times, compromises among feminist researchers over the use of gender in policy and practice and most importantly over the struggles to settle and define what gender is.

The aforementioned challenge relates with how inclusion and participation is carried out within programming and policy. According to Mary Thompson-Hall, there is a need: *to be aware of largely unsupported assumptions that inform many current policies, programs and interventions that are focused on or inclusive of gender dimensions. Especially those addressing issues for men and women through broad generalizations*. Such generalizations risk women and other marginalized groups being portrayed solely as homogeneous groups of vulnerable victims, a portrayal that can lead to misleadingly simplified solutions that could end up overlooking these groups’ capacities as agents of change, and could unintentionally result in increasing burdens of the most vulnerable. In this line of argument, Resurrección (2013) warns against essentializing women’s agency in environmental policies because it materializes understandings of gender as a fact instead of on thinking of gender as fluid, relational, contested and negotiated. Further, there are risks of increasing inequalities in gender relations, workloads and distribution of costs and risks when women’s agency is essentialized. As pinpointed by Philippa Cohen and colleagues: *traditionally, emergency aid and development interventions have tended to be single dimensional in their approaches to building adaptive capacity i.e., they focus on the delivery of assets as a means to respond to shock or to ‘fix’ complex and diverse problems within socio*-*ecological systems. The efficacy of these reactive or ‘asset*-*only’ approaches in reducing vulnerability and bringing lasting improvements to well*-*being are limited as asset*-*only approaches neglect to acknowledge other dimensions that may be enabling or inhibiting people to anticipate and respond to change*. Isabel Diaz-Reviriego, based on her research on traditional and local medical systems in Bolivia, points out that when programming and policies do not provide equal footing for women’s cultural beliefs and expertise, this can: *have significant impact on women’s authority at a local level since their responsibilities as household and community caregivers and healers could be undermined if they are not actively involved in these initiatives or if their knowledge and expertise is neglected in such strategies. Thus, if not carefully tailored, these initiatives, could compromise people’s ability to choose culturally relevant health care options, and also their adaptive capacity and health sovereignty.*

Thompson-Hall et al. (2016) highlight that feminist and gender studies hold valuable tools to move beyond these assumptions towards more tailored and meaningful understandings of the diverse vulnerabilities and adaptive capacities of different people living in the Global South. They suggest directing more time and resources in very targeted ways to apply intersectional approaches as a more effective means for addressing specific adaptation and resilience challenges. Such work embraces the complexity presented by the challenging task of integrating feminist approaches with GEC science in the pursuit of more effective adaptation strategies in the future.

## Ways ahead: Insights for the research agenda

Intersectionality emerged out of a growing recognition that it is not possible to separate out categories of class, race, ethnicity, caste, and sexuality and age nor to explain inequalities through a single framework (Valentine 2007). This special issue has explored intersections of gender and class (Buechler 2016a) as well as gender and place, class and caste (Ravera et al. 2016) in different socio-environmental contexts. However, the contributions leave a number of unexplored issues such as a deeper enquiry of race/ethnicity (Mollett and Faria 2013) and to address sexuality, ability/disability and urban contexts, which, we argue, are worthy of further analysis in GEC research. In the following lines, we would like to point at avenues of research that could enrich these conversations but acknowledging the limitations of space to deal with such a vast attempt. As Thompson-Hall suggests: *we find that ideas for how intersectionality can enrich the dialogue and body of knowledge around resilience, adaptation, and GEC is only just beginning. There are myriad ways in which this way of approaching complex identities and vulnerabilities could be integrated further into disciplines such as geography, development studies, sociology, economics, and agricultural sciences.*

Subjects described in this special issue come from rural backgrounds and rural environments that are still largely read as inhabiting heterosexual, able landscapes, where gender relations within families and communities are not only reproduced but also contested (Mortimer-Sandilands and Erickson 2010). Few studies to date analyse how resilience, vulnerability and adaptation experiences play out in different rural–urban and urban environments (for exceptions, see Ajibade et al. 2013; Jabeen 2014), gender and sexual identities and practices (but see Harcourt and Nelson 2015). For example, sexually transmitted diseases like HIV have been directly linked to demographic trends, but very geographically delimited analysis of sexuality is found in explaining interactions with natural resource management and adaptation (e.g. Béné and Merten 2008 for analyses in African countries). Overall, the intersection of gender and sexuality with other multiple identities is still nearly absent in analysis of resilience, adaptation and vulnerability. For example, Gorman-Murray et al. (2016) in their study of lesbian, gay, bisexual and trans (LGTB) experiences in Queensland floods (Australia) highlight that resilience and vulnerability are not characteristics of social groups but a product of existing societal marginality. Similarly, disability scholars have denounced the silence around people with disabilities in discourses of adaptation, mitigation, vulnerability and resilience to climate change (Wolbring 2009).

Expanding research on these areas entails more than the study of people of colour, LGTB and people with disabilities just as feminist scholarship extends beyond women to critically analyse the gender system. It entails re-thinking notions of vulnerability, adaptation and resilience following questions posed by Cote and Nightingale (2012) and Tschakert and Tuana (2013) and advancing theories of intersectionality (Garland-Thomson 2005). For example, disability studies have critiqued also notions of resilience and vulnerability defined as a property of social groups or individuals that deem individuals circumscribed to notions of capacity and competence instead as outcomes of power relations (Hutcheon and Lashewicz 2014), and propose the disability experience of interdependence as a framework for sustainability (Leipoldt 2006; Wolbring 2009).

As suggested by (Morgan 2012), in order to promote the principles of reflexivity and reciprocity in vulnerability, adaptation and resilience studies entail to bridge fragmented disciplines and knowledge, to expand feminist theory to a larger audience and to a more diversified audience than currently, to encourage scientist to engage with feminist’s theory, to improve gender diversity competencies in GEC studies in higher education and to develop novel ways of teaching in technical curricula.
